# Construction of a TAT-Cas9-EGFP Site-Specific Integration Eukaryotic Cell Line Using Efficient PEG10 Modification

**DOI:** 10.3390/ijms26031331

**Published:** 2025-02-04

**Authors:** Shiyu Qi, Yibo Wang, Zhimei Liu, Sujun Wu, Yue Zhao, Yan Li, Shoulong Deng, Kun Yu, Zhengxing Lian

**Affiliations:** 1 Beijing Key Laboratory for Animal Genetic Improvement, National Engineering Laboratory for Animal Breeding, Key Laboratory of Animal Genetics and Breeding of the Ministry of Agriculture, College of Animal Science and Technology, China Agricultural University, Beijing 100193, China; bs20223040372@cau.edu.cn (S.Q.); 2021304030309@cau.edu.cn (Y.W.); s20193040539@cau.edu.cn (Z.L.); amywsj@cau.edu.cn (S.W.); zhaoyuetx@cau.edu.cn (Y.Z.); 2 Laboratory Animal Center, Academy of Military Medical Sciences, Beijing 100071, China; scauly@cau.edu.cn; 3 National Center of Technology Innovation for Animal Model, National Human Diseases Animal Model Resource Center, National Health Commission of China (NHC), Key Laboratory of Comparative Medicine, Institute of Laboratory Animal Sciences, Chinese Academy of Medical Sciences and Comparative Medicine Center, Peking Union Medical College, Beijing 100021, China; dengshoulong@cnilas.org

**Keywords:** CRISPR/Cas9, HDR, PEG10, KI efficiency, TAT

## Abstract

The CRISPR/Cas9 system enables precise and efficient modification of eukaryotic genomes. Among its various applications, homology-directed repair (HDR) mediated knock-in (KI) is crucial for creating human disease models, gene therapy, and agricultural genetic enhancements. Despite its potential, HDR-mediated knock-in efficiency remains relatively low. This study investigated the impact of 5′ end PEG10 modification on site-specific integration of the target gene. The HEK293 cell line is considered a highly attractive expression system for the production of recombinant proteins, with the construction of site-specific integration cell lines at the AAVS1 locus enabling stable protein expression. This study investigated the impact of the 5′ end PEG10 modification on the site-specific integration of the target gene at the AAVS1 locus in the 293T cell line. Utilizing this 5′ end PEG10 modification resulted in a 1.9-fold increase in knock-in efficiency for a 1.8 kb target fragment, improving efficiency from 26% to 49%. An optimized system was utilized to successfully establish a high-expression, site-specific integration 293T cell line for TAT-Cas9-EGFP, providing a reliable resource of seed cells for subsequent protein production.

## 1. Introduction

The CRISPR/Cas9 genome-editing technology enables precise and efficient modification of eukaryotic genomes, representing a pivotal breakthrough in the field of gene editing research [1]. This system comprises the Cas9 nuclease and a bifunctional single-guide RNA (sgRNA), which directs the Cas9 protein to a complementary target site adjacent to a 5′-NGG protospacer adjacent motif (PAM) sequence. The binding of the PAM and the corresponding target site activates the Cas9 nuclease, leading to the generation of site-specific double-strand breaks (DSBs) within the genomes of diverse eukaryotic species [2,3].

DSBs induced by CRISPR/Cas9 are typically repaired via two primary DNA damage repair (DDR) pathways: the error-prone non-homologous end joining (NHEJ) pathway or the more accurate homology-directed repair (HDR) pathway. The HDR pathway utilizes a homologous chromosome or an exogenous DNA template present at the DSB site to achieve precise deletions, insertions, or point mutations. This precise modification is crucial for accurate genome editing. However, the efficiency of HDR tends to be exceedingly modest [2,4]. Hence, there remains a profound interest in further refining CRISPR/Cas9-mediated precise genome editing.

Multiple strategies have been developed to enhance the efficiency of CRISPR-Cas9-mediated HDR. Recent studies indicate that the optimization of the donor DNA template design can enhance HDR efficiency [5,6]. Specifically, the use of linearized donor plasmids has been demonstrated to significantly increase knock-in (KI) efficiency when compared to supercoiled plasmids [7]. Research has demonstrated that in vitro linearized donor templates exhibit higher KI efficiency compared to circular donors and achieve high KI efficiency across a range of cell lines [8,9,10]. Additionally, during CRISPR-Cas9-mediated HDR, gene expression from linear plasmid DNA inhibited when it is in its free intracellular form, thereby ensuring that the cells selected through positive screening with antibiotic resistance markers are primarily those in which the exogenous gene has been successfully integrated into the genomic DNA [11]. Thus, the use of linearized donor templates increases the likelihood of generating stable transfectants suitable for in vivo experimentation. Moreover, the efficiency of HDR can be markedly enhanced by chemical modifications to the donor DNA. The PEG10 modification, which involves attaching polyethylene glycol with functional groups, is commonly used to extend the half-life of protein drugs in the body [12]. This modification improves the stability of donor DNA by altering the termini of the double-stranded template [12]. A study comparing various chemical modifications revealed that donor templates modified with the chemical group polyethylene glycol (PEG10) significantly enhanced knock-in (KI) efficiency, showcasing a 5- to 6-fold improvement over unmodified controls. Additionally, these modified templates exhibited enhanced stability and reduced cytotoxicity [12].

The Cas9 RNP, also referred to as the Cas9 ribonucleoprotein complex, represents an advanced gene editing technique. The formation of the Cas9 RNP complex is crucially dependent on the incubation of the Cas9 protein with in vitro transcribed gRNA. Following introduction into the target cells, the complex serves to facilitate the gene editing process [13]. The adoption of the Cas9 RNP technique confers numerous benefits. Primarily, it minimizes the likelihood of exogenous gene integration into the host genome, thereby enhancing the safety of gene editing [14,15]. Additionally, enhancing the stability of guide RNAs (gRNAs) can mitigate off-target effects that may occur during CRISPR-Cas9-mediated gene editing and this refers to the erroneous editing of genes outside the target sites [16]. The technique is notable for its simplicity and rapidity. However, challenges remain in the direct delivery of CRISPR/Cas9 nucleases and their associated sgRNAs into cells [17]. Cell-penetrating peptides (CPPs), brief sequences of fewer than 30 amino acids, can facilitate the entry of proteins, nucleic acids, and other molecules into cells through energy-dependent endocytic pathways [18,19]. Studies have demonstrated that the fusion of Cas9 with a peptide sequence, termed Cas9-m9R, which consists of four glycine, four arginine, and four leucine residues, along with sgRNA-9R, is capable of editing endogenous genes in human cells without the need for auxiliary delivery agents [20]. Additionally, the attachment of multiple nuclear localization signal (NLS) motifs to Cas9 has been demonstrated to enhance the protein’s entry into the cell nucleus [21]. The peptide-assisted genome editing (PAGE) CRISPR-Cas system, which combines the cell-penetrating peptide HIV Transactivator of Transcription (TAT) with NLS, provides an effective method for the direct delivery of Cas9 protein, thereby supporting the successful implementation of gene editing [17].

In this study, we utilized a 5′-terminal PEG10 modification to achieve targeted gene integration, resulting in significantly improved integration efficiency compared to unmodified linear templates. Utilizing a 1.8 kb 5′-PEG10-modified double-stranded DNA (dsDNA) donor increased knock-in (KI) efficiency from 26% to 49%. This modification resulted in a 1.9-fold increase in KI efficiency relative to the unmodified dsDNA template. Additionally, we optimized a system to establish a 293T cell line with robust expression of TAT-Cas9-EGFP at the targeted integration site, thereby establishing a platform for protein production. Additionally, the expressed TAT-Cas9 protein exhibited a significant level of editing efficiency.

## 2. Results

### 2.1. PEG10 Can Enhance the Targeted Integration KI Efficiency of Target Gene at the AAVS1 Locus

Lipofection efficiency is pivotal for the creation of recombinant protein-producing cell lines. We optimized various lipofection parameters in HeLa cells, including the DNA-to-reagent ratio, amount of DNA, and incubation conditions, which significantly improved lipofection efficiency. The maximum efficiency was observed when using a complete serum-containing medium devoid of antibiotics, with a DNA-to-liposome ratio of 1:1.5 (Figure S1A,F). However, a simultaneous increase in the quantities of both DNA and liposomes did not enhance transfection efficiency (Figure S1B,G). Additionally, we applied these optimized conditions to HEK 293T cells, which are particularly suitable for gene editing studies and the production of recombinant proteins. The lipofection efficiency in 293T cells was significantly higher than that in HeLa cells (Figure S1H,I). For co-transfection with pX458 and the donor, the optimal mass ratio was 1:1.5, corresponding to a molar ratio of 1:3.75, which yielded the highest co-transfection efficiency (Figure S1D,J). Utilizing the pX458 plasmid (9288 bp), the transfection efficiency in 293T cells reached 50.7 ± 1.7% (Figure S1C,H), while co-transfection with the donor template achieved an efficiency of 14.3 ± 0.5% (Figure S1E,I). The *AAVS1* site, located on chromosome 19 within the first intron of the *PPP1R12C* gene (protein phosphatase 1 regulatory subunit 12C), is a well-characterized genomic safe harbor (GSH) [22,23]. To selectively target the *AAVS1* locus in 293T cells, we selected an optimized sgRNA for site-specific cleavage (Figure S2A). The efficacy of the sgRNA was validated through T7EI nuclease assay and sequencing (Figure S2B,C). To assess the potential for off-target effects of Cas9 in the 293T genome, we identified potential off-target sequences for the sgRNA [24]. These sequences are characterized by a preceding NGG motif and exhibit the highest homology to the guide RNA. Subsequently, we analyzed six potential off-target sites with increased probability (Figure S2D). No mutations or indels were observed upon alignment with NCBI reference sequences, suggesting the absence of off-target effects in this cell line.

**Figure 1:**
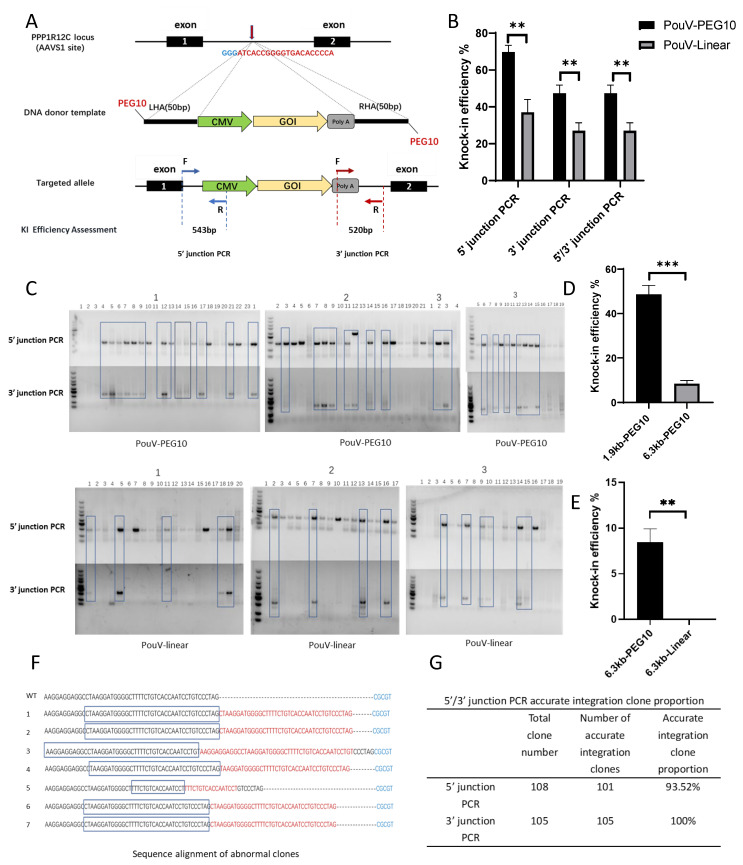
PEG10 modification can significantly enhance the knock-in (KI) efficiency of genes. (**A**) Schematic diagram of the targeting strategy for the 50 bp HA knock-in of the AAVS1 site with PEG10-modified exogenous DNA fragment. The repair template sequence from 5′ to 3′ consists of 5′ homology arm sequence, CMV promoter, target gene sequence, SV40 polyA transcription termination sequence, and 3′ homology arm sequence. The blue and red arrows represent the upstream and downstream primers for the 5’ junction PCR, respectively. (**B**) KI efficiency of stable cell clones (cPouV). The KI efficiency was obtained by the percentage of 5′/3′ junction PCR positive clones among the total clones investigated post-transfection, wherein we separately counted the ratios of positive clones at the 5′ end, 3′ end, and both sides of the junction to the total number of clones. *n* = 3 biological replicates; ** indicates *p* < 0.01. (**C**) Electrophoresis diagram showing the insertion of the exogenous DNA template cPouV detected by 5′/3′ junction PCR, with amplification bands of 543 bp and 537 bp in size. Groups 1, 2, and 3 represent the three different groups, respectively. (**D**) KI efficiency of fragments with different lengths modified with PEG10. *n* = 3 biological replicates; *** indicates *p* < 0.001. (**E**) KI efficiency of stable cell clones (TAT-Cas9-EGFP). *n* = 3 biological replicates; ** indicates *p* < 0.001. (**F**) Sequence alignment of abnormal clones. (**G**) Sequencing results showed the proportion of clones with accurate integration clonesall clones.

We targeted the genes *cLin28A*, *cLin28B*, *cNanog*, and *cPouV*, which are integral to the maintenance of pluripotency in embryonic stem cells (ESCs) [25,26,27,28]. The HDR template sequence consists of the following elements from 5′ to 3′: a 5′ homology arm sequence, the CMV promoter, the target gene sequence, the SV40 polyA transcription termination sequence, and a 3′ homology arm sequence, with PEG10 modification (Figure 1A). The lengths of the four homology repair templates are 1565 bp, 1685 bp, 1910 bp, and 1844 bp, respectively. The KI efficiency for each group was calculated as the percentage of ligation PCR-positive clones among the recovered single clones (based on positive PCR amplimers for both 3′ and 5′ ligation PCR). The results of the 5′/3′ junction PCR showed that using a 1.8 kb 5′ PEG10-modified dsDNA donor (PouV) at the *AAVS1* locus in HEK293T cells increased KI efficiency from 27% to 47% (Figure 1B,C). When a 1.5 kb 5′ PEG10-modified dsDNA donor (Lin28A) was used, the KI efficiency could be increased from 28% to 53% (Figure S3A). Utilizing the 5′ PEG10-modified dsDNA as a template resulted in a 1.9-fold increase in KI efficiency compared to the unmodified template (Table S7). Similarly, targeted KI was effectively achieved in the cLin28B and cNanog target genes as well. The KI efficiency of cLin28B was approximately 53%, and cNanog was 49% (Figure S3B). Furthermore, within the AAVS1 locus of 293T cells, the integration of a 6.3-kilobase fragment (TAT-Cas9-EGFP) resulted in a knock-in efficiency of 8.82% when PEG10 modification was employed. In contrast, the absence of PEG10 modification yielded no positive clones. (Figure 1D,E). This result indicates that PEG10 is capable of integrating larger DNA fragments effectively.

The sequencing of the PCR-amplified fragments confirmed the successful insertion of the exogenous genes (*cLin28A*, *cLin28B*, *cPouV*, and *cNanog*) into the target sites. All 105 amplified 3′ PCR fragments analyzed exhibited accurate homologous recombination. The accuracy rate of homologous recombination within the 108 analyzed 5′ PCR fragments was 93.52% (Figure 1F). Among the 108 amplified 5′-end PCR fragments analyzed, four abnormal integration fragments were identified, each displaying a distinct integration pattern (Figure 1E). These abnormal fragments contained sequences with repeated the homology arms, suggesting that homologous recombination likely involved fragments less than 50 bp in length serving as homology arms. The frequency of abnormal integration fragments was only 3.81%, which is negligible compared to the overall results.

The *AAVS1* locus was also selected in HeLa cells, and a 5 kb (5004 bp) homologous recombination template was engineered with 5′ PEG10 as a modification. From the screening of twelve cell clones, one was identified as a positive clone, resulting in an efficiency of 8.33%. This result indicates that the PEG10 modification is also effective for use in HeLa cells. (Figure S4A–C). Given the inherently low HDR efficiency in primary fibroblasts [29,30], we also evaluated editing efficiency in goat fetal fibroblast (GFF) cells. A 109 bp homology repair template, modified with 5′ PEG10, was utilized, possessing homology arms that were 500 bp in length and specifically targeted the regulatory region of the *lactoferrin* gene (Figure S5A). The screening of 33 cell clones yielded one positive clone, corresponding to a positive rate of 3.03%. In contrast, with the unmodified conventional linear homology repair template, 48 cell clones were tested, but no positive clones were identified (Figure S5C). Only one positive clone was identified from the 33 generated clones, which is considered low efficiency. This low efficiency is suspected to be related to the diminished editing efficacy at the target locus. To address this, an additional sgRNA was introduced that overlaps with the original sgRNA [31]. Following this modification, a total of 62 clones were generated, of which 16 were positive, resulting in a positive rate of 25.8% (Figure S5B,C).

In summary, the 5′ PEG10-modified dsDNA donor increased knock-in efficiency by 1.8-fold, achieving a maximum KI rate of 49% in 293T cells. This method is also effective in Hela and goat fibroblast cells.

### 2.2. Establishment of a Site Specifically Integrated Four Factors Cell Lines

**Figure 2:**
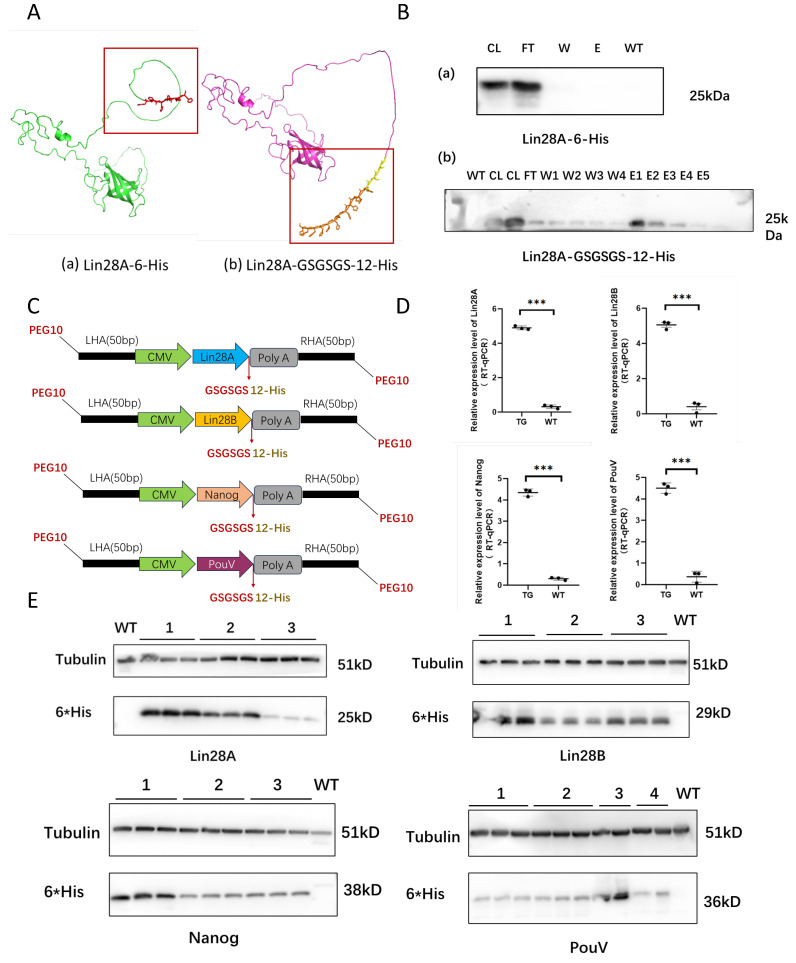
Establishment of a site specifically integrated four factors cell lines. (**A**) Predicted three-dimensional structure of Lin28A protein. (**a**) Three-dimensional structure diagram of Lin28A-6 His. (**b**) Three-dimensional structure diagram of Lin28A-linker-12 His. (**B**) Western blot results of purification of His-tagged recombinant proteins. (**a**) Purification results before optimization. (**b**) Purification results after optimization. WT, wild type; CL, cell lysis fraction; FT, flow through fraction; W, wash fraction; E, elution fraction. (**C**) Schematic diagram of homologous recombination templates for Lin28A, Lin28B, PouV, and Nanog. (**D**) qPCR detection of mRNA expression levels of Lin28A, Lin28B, PouV, and Nanog TG, Target genes; WT, wild-type; *n* = 3 biological replicates; *** indicates *p* < 0.001. (**E**) Western blot detection of protein expression levels of Lin28A, Lin28B, PouV, and Nanog. The numbers 1, 2, 3, and 4 each represent different clones, with each clone undergoing three technical replicates. 6*His represents the 6-his tag.

Cell lines with site-specific integration of four factors, each modified with PEG10 and carrying *cLin28A*, *cLin28B*, *cPouV*, and *cNanog*, respectively, have been developed. Despite efforts, protein purification did not yield adequately purified protein. The difficulties are thought to be associated with the tags and linker of the recombinant protein. To address this, a GSGSGS linker was introduced, and the number of His tags was increased from six to twelve. Predicted three-dimensional structure analysis revealed that these modifications not only maintained the native structure of Lin28A but also improved the tag’s exposure, thereby enhancing its affinity for Ni^2+^ (Figure 2A). Western blot (WB) results indicated that the use of a 6 His tag without a linker resulted in proteins being retained in the flow-through fraction, implying insufficient binding to Ni^2+^ and precluding effective purification. However, the incorporation of the GSGSGS linker and the increase in His tags from 6 to 12 significantly increased the presence of the recombinant protein in the elution fraction, with minimal amounts found in the flow-through and wash fractions, thus greatly improving purification efficiency (Figure 2B). The optimization of the linker prevents misfolding of the fusion protein and alters its spatial conformation. Increasing the His tag from 6 to 12 enhances specificity and facilitates more effective purification of the recombinant protein.

Next, using the optimized system, cell lines with site-specific integrations of *cLin28A*, *cLin28B*, *cPouV*, and *cNanog* were prepared (Figure 2C). To assess the stability of expression, three or four clones from each cell type were randomly selected for analysis. Quantitative PCR (qPCR) analysis revealed significant mRNA expression for all four cell types (Figure 2D). Subsequently, total protein extracts from cell lysates expressing *cLin28A*, *cLin28B*, *cPouV*, and *cNanog* were subjected to Western blot analysis using an anti-6 His antibody. Target bands at approximately 25, 29, 38, and 36 kDa were detected in the respective cell lines, whereas no specific bands were observed in the wild-type 293T cell samples (Figure 2E). The data confirm the stable expression of *cLin28A*, *cLin28B*, *cPouV*, and *cNanog* at both the transcriptional and translational levels. Notably, however, protein expression levels vary across different cell lines, despite comparable mRNA expression. The establishment of cell lines that robustly express recombinant Lin28A, Lin28B, Nanog, and PouV underscores the efficacy and flexibility of the optimized system.

### 2.3. Establishment of a Site Specifically Integrated TAT-Cas9-EGFP Cell Line

**Figure 3:**
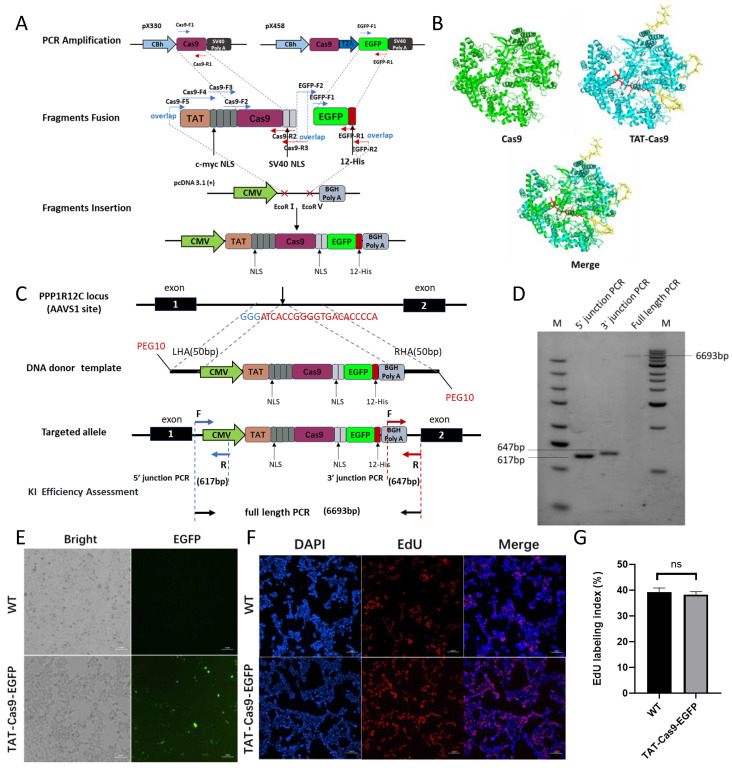
Establishment of a site specifically integrated TAT-Cas9-EGFP cell line. (**A**) The construction process of the TAT-Cas9-EGFP donor plasmid. The elements from 5′ to 3′ are as follows: TAT, 4-NLS, Cas9 protien, 2-NLS, EGFP, 12 His. Blue arrow above the element: Upstream primer; Red arrow below the element: Downstream primer; Dashed lines within the primer sequences: Overlap sequences for seamless cloning. The red cross symbol represents the restriction enzyme cutting sites EcoR I and EcoR V. (**B**) Three-dimensional structure prediction results of TAT-Cas9 protein. The figure on the left shows the three-dimensional structure of the Cas9 protein. The figure on the right shows the predicted three-dimensional structure of the TAT-Cas9 protein. The figure below shows the result of the merging of the two. The red area represents TAT, and the yellow area represents NLS. (**C**) Schematic of TAT-Cas9-EGFP targeted integration and PCR validation. The first part is a diagram of sgRNAs at the AAVSI site, with the CRISPR RNA (crRNA) sequences shown in red font and the PAM sequences in blue. The second part is the template structure, with the elements from 5′ to 3′ as follows: TAT, 4-NLS, Cas9 protein, 2-NLS, EGFP, 12-His. The third part is the identification of targeted integration. The blue arrow indicates the primer for 5′ junction PCR, the red arrow indicates the primer for 3′ junction PCR, and the black arrow indicates the primer for full-length PCR. (**D**) Electrophoresis diagram showing the detection of positive clones by PCR. From left to right, they are marker, the 5′ end site-specific integration result with T-Left-F/R, the 3′ end site-specific integration result with T-Right-F/R, and the full length result with T-Left-F and T-Right-R. (**E**) The morphological structures of TAT-Cas9-EGFP cells and WT cells were examined under the microscope. Scale bar 100 μm. (**F**) EdU staining of TAT-Cas9-EGFP cells and WT cells. Scale bar 100 μm. (**G**) The EdU labeling index, represented as the number of EdU-positive cell nuclei/total cell nuclei.

**Figure 4:**
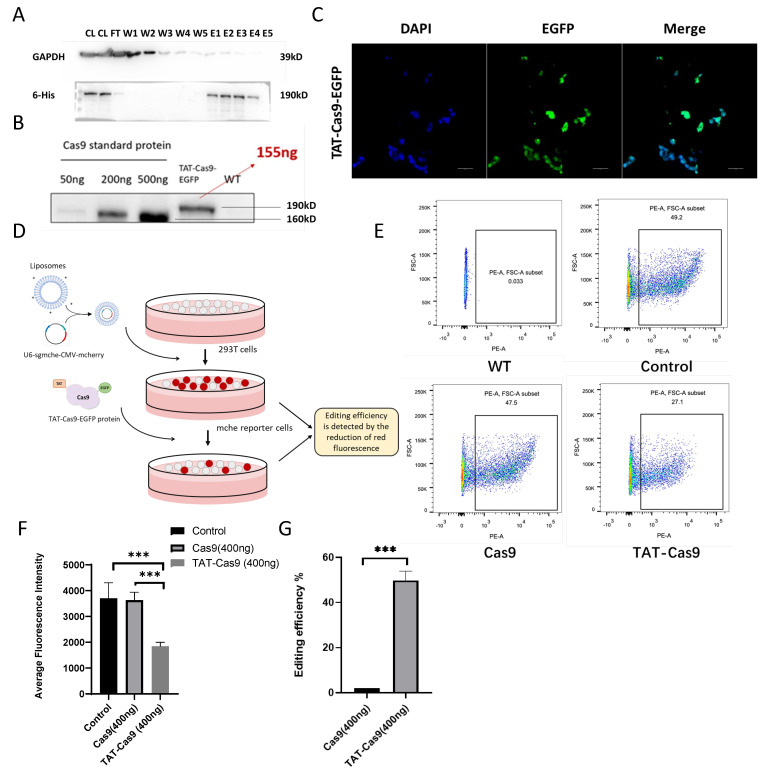
Validation of TAT-Cas9-EGFP Protein Activity. (**A**) Western blot results of purification of His-tagged recombinant proteins. CL, cell lysis; FT, flow through; W, wash; E, elution. (**B**) Western blot, and the protein recovery was estimated by comparing the gray values with the standard Cas9 protein. (**C**) The results of observing the entry of TAT-Cas9-EGFP under a confocal microscope. Scale bar 50 μm. (**D**) Schematic workflow for quantifying gene editing efficiency using 293T mCherry (mChe) reporter cells. Stable expression of mChe fluorescent reporter gene was achieved using a bicistronic expression vector, and targeted mCherry gene was used for liposome transfection. 293T cells were incubated with TAT-Cas9-EGFP protein, and after 48 h, gene editing efficiency was quantified by measuring the loss of mChe-positive cell populations using flow cytometry. Blue represents liposomes, red represents TAT, purple represents Cas9, and green represents EGFP. (**E**) Calculation of average fluorescence intensity by FACS. (**F**) The results of mean fluorescence intensity. (**G**) Editing efficiency. Gene editing efficiency was assessed by comparing the reduction in mean fluorescence intensity compared to the control group. *** indicates *p* < 0.001; The experiment was performed in at least two replicates.

TAT, a peptide class known for its ability to traverse the cell membrane, when combined with Nuclear Localization Signals (NLSs), facilitates the direct delivery of Cas proteins [17]. Subsequently, a site-specific integration cell line, TAT-Cas9-EGFP, was successfully developed through the integration of a PEG10 modification. The HDR template is defined by the following components: a CMV promoter, the cell-penetrating peptide TAT, six nuclear localization signals (NLS), EGFP, a 12 His (histidine tag) motif, and a BGH polyA, encompassing a total length of 6309 base pairs [17] (Figure 3A). AlphaFold 3 predictions suggested that the incorporation of TAT and NLS sequences does not compromise the structural integrity of the Cas9 protein (Figure 3B). The *AAVS1* locus in 293T cells was targeted for HDR (Figure 3C). Among the 68 clones analyzed, 6 positive clones were detected, and the clone with the most robust growth was selected for subsequent experiments. These positive clones displayed green fluorescence under fluorescence microscopy (Figure 3E). Genomic DNA was extracted and subjected to PCR amplification of the 5′/3′ junctions and full-length gene, followed by sequencing to confirm site-specific integration (Figure 3D). TAT-Cas9-EGFP cells were found to be morphologically identical to WT cells (Figure 3E). An EdU cell proliferation assay further demonstrated that TAT-Cas9-EGFP cells and WT cells had comparable proliferative capacities (Figure 3F,G). The findings suggest that no adverse cellular alterations were detected post-integration, underscoring the efficacy and potential of the insertion strategy. Western blot analysis confirmed the successful purification of the target protein (Figure 4A). The yield of purified protein was estimated to be approximately 3.72 µg from a population of 1.5 × 10^7^ cells. (Figure 4B). Upon addition to 293T cells, the purified TAT-Cas9 protein is effectively transported into the cell nucleus (Figure 4C). A plasmid construct was engineered to encode a red fluorescent protein, mCherry, along with an sgRNA directed against mCherry. This construct was introduced into 293T cells via liposome-mediated transfection, while simultaneously incubating the cells with TAT-Cas9-EGFP protein (Figure 4D). Flow cytometry analysis conducted 48 h post-transfection (Figure 4E) revealed an editing efficiency of 50%, as assessed by the decrease in red fluorescence intensity relative to the control (Figure 4F,G). In conclusion, a cell line with site-specific integration of TAT-Cas9-EGFP has been successfully established, and the TAT-Cas9-EGFP protein exhibits stable expression, thereby providing a reliable system for protein production.

## 3. Discussion

For the precision of genome editing, double-strand breaks (DSBs) are typically repaired via the homology-directed repair (HDR) pathway, which is most active during the late S/G2 phases of the cell cycle [32]. HDR is pivotal for advancements in animal breeding; however, its naturally low efficiency poses a substantial obstacle for the application of CRISPR-Cas9 in targeted genome modification [29,30]. One major factor limiting HDR efficiency is the structure of the repair template [6,9]. Studies have demonstrated that linearized plasmids can enhance KI efficiency when using CRISPR/Cas9 technology [7,8,9,10]. Moreover, linearized vectors tend to yield more stable transfected cells, which are more appropriate for in vivo animal studies [11]. Consequently, this study utilized linearized vectors for HDR to generate site-specific integration cell lines. In this study, the repair template was a linear DNA fragment with a 5′ C6-PEG10 modification. This modification serves to inhibit the concatenation of multiple templates, an occurrence that can lead to the insertion of multiple DNA fragments into the genome when using CRISPR/Cas9 technology [33]. Furthermore, this approach reduces the likelihood of partial DNA fragment loss during the editing process. The attachment of molecules to the termini of the DNA template aids preventing unintended ligation, minimizing erroneous DNA insertions, and guaranteeing the comprehensive integration of the DNA into the genome [34,35]. Additionally, the 5′C6-PEG10 modification shields the DNA termini from degradation by cellular nucleases, thereby enhancing DNA stability and elevating the probability of nuclear import and chromosomal integration [12]. A recent investigation evaluated the efficacy of diverse chemical modifications and revealed that modification of the 5′ end with a C6-polyethylene glycol (C6-PEG10) moiety significantly enhances KI efficiency, while also demonstrating high stability and reduced cytotoxicity [12]. The incorporation of the modification has demonstrated an enhancement in stability, ensuring that the gene editing process is maintained with a certain level of precision, without concomitantly increasing the incidence of random transgene insertion. Insertions and deletions (indels) at the KI junction regions are commonly a result of non-homologous end joining (NHEJ)-mediated KI, whereas HDR-mediated KI typically results in accurate editing [36]. The PEG10 modification does not elevate the NHEJ-KI frequency, thereby maintaining a low rate of random integration events [12]. The previous study validated the knock-in (KI) efficiency enhancement by PEG10 at various loci including AAVS1, GAPDH, and CCR5 in 293T cells, consistently demonstrating that PEG10 significantly increases KI efficiency [12]. Compared to other safe harbors, the AAVS1 locus has high and uniform gene expression [22,23], which is why the AAVS1 locus was chosen for subsequent experimental research in 293T cells. Experimental data indicate that the utilization of a 1.8 kb dsDNA donor with 50 bp homology arms and a 5′ PEG10 modification at the AAVS1 locus in HEK293T cells augmented KI efficiency from 26% to 49%, corresponding to a 1.9-fold enhancement. This chemical modification bolstered DNA stability and promoted nuclear import. Additionally, incorporating a 50 bp homology arm within PCR primers enables expedited template synthesis through straightforward PCR and diminishes the length of the linear repair template, which may enhance transfection efficiency. The effectiveness of the PEG10 modification for KI of large fragments (6.3 kb) is validated by the establishment of the TAT-Cas9-EGFP cell line, which demonstrates an editing efficiency of 8.82%. However, the efficiency is diminished for extended fragments, a phenomenon that can be attributed to reduced transfection efficacy accompanying increased fragment size [37] and the complexities inherent in integrating larger DNA segments [38].

Genetically modified animals with KI transgenes play a significant role in the genetic improvement of livestock. The efficiency of HDR in primary fibroblasts is inherently low [29,30], posing significant challenges for targeted genome modifications using CRISPR/Cas9. The addition of PEG10 modification contributes to homologous recombination in goat fibroblasts. In GFF cells, we utilized a 500 bp homology arm length [39], and incorporated a PEG10 modification, resulting in a KI efficiency of 3.03%. This marks a considerable enhancement over the efficiency observed with unmodified templates. Despite screening 33 clones, only one positive clone was identified, indicating an exceptionally low success rate. The diminished efficiency is hypothesized to correlate with the reduced efficacy of editing at the target locus. Research has indicated that the employment of multiple sgRNAs with overlapping sequences can improve the efficiency of CRISPR/Cas9-mediated knock-in. Consequently, an additional sgRNA was introduced, which overlaps with the initial sgRNA. Following this modification, we isolated a total of 62 clones, of which 16 were positive, resulting in a knock-in success rate of 25.8%. Future research endeavors should concentrate on refining HDR efficiency through various approaches, such as optimizing the length of the homology arm [40], and incorporating small molecule inhibitors [41], in order to achieve more precise and effective knock-in results. Prokaryotic expression systems are advantageous for protein production due to their convenience and numerous benefits. Nonetheless, proteins synthesized in these systems may exhibit reduced biological activity and frequently necessitate additional modifications for functionality. Furthermore, prokaryotic systems lack the ability to finely regulate expression timing and levels, and certain proteins may accumulate as inclusion bodies, which can complicate the purification process. In comparison, mammalian cell lines are increasingly favored for the production of recombinant proteins, as they are capable of executing the necessary post-translational modifications (PTMs) [42]. HEK293 cells are particularly beneficial for recombinant protein production due to their ability to generate human-like glycosylation patterns, thereby minimizing the risk of immunogenic reactions [43,44,45]. Additionally, these cells facilitate the stable yield of recombinant proteins [43,46,47]. Genomic safe harbors (GSHs) are discrete genomic loci that serve as optimal insertion sites for the stable expression of exogenous proteins without interfering with the function of other genes [22]. The AAVS1 locus, situated within the first intron of the PPP1R12C gene on chromosome 19, is a well-established GSH. The targeting of this site does not impair cellular function and supports the consistent transcription and stable expression of the inserted genetic material [22]. The AAVS1 site is characterized by high and uniform levels of transgene expression, maintaining stability for approximately three months without the requirement for ongoing selection pressure [23]. Traditionally, the development of recombinant protein-producing cell lines involves the random integration of the target gene into the host genome, followed by a laborious screening process to isolate clones with high expression levels [48,49]. This methodology is not only time-intensive, often extending over a year, but also economically burdensome. Furthermore, the random integration strategy can result in variable expression stability and diminished yields over time, attributable to the unpredictable nature of integration sites and potential genetic instability [50,51]. To overcome these challenges, site-specific gene integration technology enables the accurate placement of the target gene within transcriptionally active genomic regions [52]. Targeting these regions for site-specific integration expedites the creation of high-expression recombinant protein cell lines and obviates the unpredictability inherent in random integration methods [53,54]. The generation of site-specific integration cell lines at the AAVS1 locus in 293T cells ensures stable protein expression, furnishes a dependable source for protein production, and lays the groundwork for subsequent experimental procedures. Affinity chromatography, a standard method for purifying recombinant proteins, typically involves the binding of a 6 His tag to nickel-charged NTA ligands. However, certain proteins may exhibit non-specific binding to nickel-based resins, which can lead to reduced yields of 6 His-tagged recombinant proteins. In a study, nickel resin was used to purify rSHI overexpressed by Pseudomonas pelliculosa, and it was found that using a 12 His tag could enhance the affinity between the protein and the nickel resin, making it easier to purify, and no significant amounts of non-specific proteins were observed in any elution [55]. In this study, a eukaryotic cell line with site-specific integration was established, and the purification of recombinant proteins was achieved through the utilization of an N-terminal 12 His tag. The results indicated that increasing the number of His tags from 6 to 12 further improved specificity, thereby facilitating the purification of the recombinant protein. Furthermore, the incorporation of a linker-tag was found to maintain the protein’s native conformation and was demonstrated to be a versatile strategy for the purification of diverse proteins.

The direct transduction of CRISPR-Cas9 nucleases and their corresponding sgRNAs into cells is frequently hindered by inefficient cellular internalization, attributable to the absence of inherent cell-penetrating properties. A study has shown that Cas9 nucleases, when complexed with polycationic sgRNAs, can associate with cationic liposomes, which enhances their delivery efficiency into mammalian cell lines [56]. However, components like liposomes can be toxic, highlighting the importance of optimizing direct protein delivery methods. Studies have shown that Cas9, when chemically conjugated with polyarginine peptides, can be internalized by cells through endocytosis [20]. Furthermore, the addition of multiple nuclear localization signals (NLSs) to Cas9 has been shown to improve its delivery into the cell nucleus [21]. The Peptide-Assisted Genome Editing (PAGE) CRISPR-Cas system, which comprises core components such as TAT, Cas proteins (including Cas9 or Cas12a), multiple nuclear localization signals, and endosome escape peptides, provides a simple, efficient, and non-toxic approach to genome editing in primary cells [17]. In contrast to the prokaryotic expression system detailed in the aforementioned publication, a eukaryotic expression system has been constructed for the specific integration of TAT-Cas9-EGFP, leading to the establishment of stable protein expression. 

## 4. Materials and Methods

### 4.1. Plasmid Construction

The Cas9 and U6-sgRNA co-expression vector backbone pX458 was purchased from Addgene (plasmid ID: 48138). The sgRNA(ACCCCACAGTGGGGCCACTA) was designed using the CRISPR design tool (https://www.ensembl.org/index.html accessed on 13 December 2024) based on the target site at the AAVS1 locus. To construct the Cas9/gRNA plasmid, the pX458 plasmid was digested with the restriction enzyme BbsI to allow for the cloning of additional sgRNAs. The sgRNA was synthesized, annealed, and cloned into the pX458 backbone vector to form a functional co-expression plasmid [57].

To construct the HR donor plasmid, an HA corresponding to the target site was designed using the human genomic DNA sequence from NCBI. The homology repair template sequence, from 5′ to 3′, consists of the following elements: a 5′ homology arm sequence, a CMV promoter, a protein-coding gene sequence (Lin28A/Lin28B/Nanog/PouV), an SV40 polyA transcription termination sequence, and a 3′ homology arm sequence. The CMV promoter and SV40 polyA sequence were obtained from the Pmcherry-N1 plasmid. PCR amplification of the Lin28A, Lin28B, Nanog, and PouV sequences was performed using the laboratory’s existing pIRES-cLin28A, pIRES-cLin28B, pIRES-cNanog, and pIRES-cPouV plasmids, respectively. Digestion of the Pmcherry-n1 plasmid with NheI and NotI restriction enzymes was followed by the cloning of the PCR-amplified fragments into the vector backbone. The donor plasmid was constructed using the ClonExpress MultiS One-Step Cloning Kit (Vazyme, Nanjing, China) according to the manufacturer’s instructions. The 50 bp HA sequence, due to its brevity, can be acquired through the design of specific primers. Modified primers with a 5′ C6-PEG10 modification were used to amplify the circular plasmid, linearizing the donor plasmid with the modification.

To generate an expression vector for the recombinant TAT-Cas9-EGFP protein expression in cells, the components of the expression vector were amplified by PCR. The integrated template sequence, from 5′ to 3′, consists of the following elements: the TAT sequence, 4-c-myc NLS, Cas9, 2-SV40 NLS, EGFP, and a 12-His tag. Cas9 was amplified from the pX330 plasmid (plasmid ID: 42230, Addgene, Watertown, MA, USA), and the elements TAT, 4-c-myc NLS, 2-SV40 NLS, as well as the 20 bp overlap necessary for seamless cloning, were incorporated into Cas9 through a series of primer design and amplification steps. The EGFP sequence was amplified from the pX458 plasmid and modified through multiple amplification steps to include the 12 His tag and the required 20 bp overlap for seamless cloning. Digestion of the pcDNA3.1(+) vector with EcoRI and EcoRV enzymes was performed, followed by the cloning of the aforementioned PCR-amplified fragments into the vector backbone. Construction of the donor plasmid was achieved using the pEASY^®^-Basic Seamless Cloning and Assembly Kit (TRAN, Beijing, China), in accordance with the manufacturer’s protocol. Acquisition of the 50 bp homology arm (HA) was facilitated by the design of specific primers, due to its brevity. Amplification of the circular plasmid was performed using modified primers containing a 5′ C6-PEG10 modification, subsequent to which the plasmid was linearized with the same modification. The primers used are listed in Table S1. The red fluorescent reporter plasmid U6-sgmChe-CBh-mcherry was designed. Its sequence from 5′ to 3′ is as follows: the U6 promoter, an sgRNA targeting mcherry, the CBh promoter, and mcherry. The sequence from 5′ to 3′ encompasses the U6 promoter, an sgRNA directed against mcherry (GGAGCCGTACATGAACTGAG), the CBh promoter, and the mcherry gene. The U6 promoter, CBh promoter, and mcherry gene were derived from the laboratory’s pre-existing pX330-mcherry plasmid. This section was amplified via PCR and subsequently cloned into the pcDNA3.1(+) vector backbone, utilizing EcoRI and EcoRV as the selected restriction enzyme sites. The plasmid was finally digested using the restriction enzyme BbsI to enable the cloning of additional sgRNAs. The primers utilized are detailed in Table S2.

All components of the plasmids were amplified using PrimeSTAR Max (Takara, Japan) under the following settings: 98 °C for 10 s, 60 °C for 15 s, 72 °C for 5 s per kilobase, for 30 cycles. All donor templates were purified using the DNA purification kit (TIANGEN, Beijing, China) and sequenced by NovaSeq Company (San Diego, CA, USA).

### 4.2. Cell Culture and Transfection

The human embryonic kidney cell line HEK 293T and Hela cell line are preserved by the Cell Resource Center of the Institute of Basic Medical Sciences, the Chinese Academy of Medical Sciences, and cultured in a cell culture incubator at 37 °C with 5% CO_2_ humidity. The 293T cells are maintained in high-glucose DMEM (Gibco, Waltham, MA, USA) supplemented with 10% fetal bovine serum (FBS, Gibco) and 1% penicillin/streptomycin (Gibco). Hela cells are maintained in 1640 medium (Gibco) supplemented with 10% FBS and 1% penicillin/streptomycin. Goat fetal fibroblast (GFF) cells are derived from the fetuses of Lasoshan dairy goats (*Capra hircus*) at 6 to 7 weeks of gestation and cultured in complete medium consisting of DMEM/F12 (Gibco), 10% FBS, and 1% penicillin/streptomycin.

Lipofect approximately 10^6^ cells with the corresponding amount of pX458-sgRNA and linear donor using Lipofectamine™ 3000 (Thermo Fisher Scientific, Waltham, MA, USA), following the manufacturer’s instructions, with the specific ratios of liposome to plasmid detailed in Table S3. After 48 h of lipofection, the cells were stained with DAPI, and images were captured using an inverted fluorescence microscope. Cell counting was performed using Image J 1.46r.

### 4.3. T7 Endonuclease I Cleavage Detection Assay

T7 Endonuclease I (T7EI) can recognize and cut small homologous repeat sequences within DNA molecules, and it can be used to quantify the efficiency of gene editing events. CRISPR/Cas9-induced lesions at the endogenous target site was quantified using the T7EN I (NEB) cleavage detection assay to investigate the insertions/deletions (indels) generated by nuclease-mediated non-homologous end joining (NHEJ). The gene fragments of off-target sites were amplified with primers specific to each locus by 30 cycles of PCR. The obtained PCR products were purified using a kit (Tiangen). A quantity of 0.5–1 μg of the purified product was denatured and annealed using a thermocycler, subsequently digested with T7EN I for 15 min and separated with a 2% agarose gel. Electrophoresis was conducted at 100 V for a duration of 30 min. The primers are listed in Table S4.

### 4.4. Lysis of Trace Cells and 5′/3′ Junction PCR at Target Region

Cells were sorted by flow cytometry into a 96-well plate 48 h post-lipofection. The cell culture medium was changed every 5 days, and when the wells of the 96-well plate were confluent, positive cell identification could be performed. The microcell lysis buffer system consists of: Tris-HCl (1 M pH = 8.0) 2 mL, Triton X-100 0.45 mL, NP-40 0.45 mL, Proteinase K (20 mg/mL) 1 mL, and sterile ddH_2_O to a final volume of 50 mL. The lysis procedure is as follows: 65 °C for 30 min; 95 °C for 15 min; 16 °C indefinitely.

The lysate product was taken at a volume of 2 μL for PCR identification. The PCR reaction mix for all samples included template DNA at 20 ng, forward and reverse primers each at 0.5 pmol, PrimeSTAR 12.5 μL, and the volume was brought to 25 μL with sterile double-distilled water. The PCR reaction conditions were as follows: pre-denaturation at 98 °C for 5 min; 30 cycles of denaturation at 98 °C for 10 s, annealing at 59 °C for 15 s, and extension at 72 °C for 5 s per kilobase; a final extension at 72 °C for 5 min; and a final hold at 4 °C. The PCR products were analyzed on a 1% agarose gel. The 5′ primer Left-F was designed outside the 5′ homology arm of the AAVS1 gene, and Left-R was designed within the CMV promoter of the insert fragment, generating a 543 bp amplification product from CRISPR/Cas9-mediated targeted integration cells. The 3′ primer Right-F was designed within the SV40 polyA sequence of the insert fragment, and Right-R was designed outside the 3′ homology arm sequence of the AAVS1 gene, generating a 537 bp amplification product from CRISPR/Cas9-mediated targeted integration cells. The purified 5′/3′ ligated PCR products were sequenced by NovaSeq Company. The detailed DNA sequences used for PCR primers are listed in Table S5.

### 4.5. Real-Time Fluorescence Quantitative PCR

To determine the relative copy numbers of genes encoding cLin28A, cLin28B, cNanog, and cPouV, total RNA was extracted using the RNeasy Micro Kit (Qiagen, Hilden, Germany) according to the manufacturer’s instructions. cDNA was synthesized using the PrimeScript RT Kit (Takara, Kusatsu, Japan) following the manufacturer’s protocol. Tubulin was used as a representative housekeeping gene for normalization. For qPCR assays, each 20 μL qPCR reaction mixture contained 2 μL of cDNA (approximately 100 ng of RNA), 0.4 μM of forward and reverse primers, 0.4 μL of Rox Reference Dye II, and 10 μL of SYBR Premix Ex Taq II (Takara). The qPCR reactions were run on an Mx3000P instrument (Agilent Technologies, Santa Clara, CA, USA) with the following conditions: 95 °C for 30 s, followed by 40 cycles of 95 °C for 5 s, 60 °C for 30 s, and 72 °C for 30 s. Data analysis was performed using Mx3000P. The relative copy numbers of endogenous target genes in wild-type 293T cells and the related transgenes in site-specific integration-derived reference clones were calculated using the ΔΔCT method: (log_10_(2^−ΔΔCT^) + 1). The detailed DNA sequences used for qPCR primers are listed in Table S6.

### 4.6. Detecting Targeted Protein Expression

After 5′/3′ ligation PCR identification, the expression titers of positive clones were further analyzed using Western blotting. Then, 2.5 × 10^6^ cells were collected, and total protein was extracted using RIPA lysis buffer (Solarbio, Beijing, China). The protein concentration of each extract was quantified using the BCA Protein Assay Kit (cwbio, Taizhou, China) according to the manufacturer’s protocols. Then, mix crude lysate in SDS buffer and incubated at 100 °C for 5 min, equal amounts of the protein samples (about 20–30 μg each) were loaded in separate lanes of 10% gel for SDS–polyacrylamide gel electrophoresis (Bio-Rad, Hercules, CA, USA). After electrophoresis, the proteins were transferred to a PVDF membrane under conditions of 4 °C, 320 mA, and 1.5 h. The PVDF membrane was blocked with 5% non-fat milk at room temperature for 2 h. After washing with TBST, the membrane was incubated with the primary antibody overnight at 4 °C. The antibody used for identification of the His tag was a 1:10,000 dilution of 6 His, His-tag monoclonal antibody (66005-1). The Cas9 antibody used was a 1:1000 dilution of the recombinant anti-CRISPR-Cas9 antibody [EPR18991] (ab189380). Following another wash with TBST, the membrane was incubated with the secondary antibody at room temperature for 1 h. The antibodies are all diluted in TBST. Finally, the target proteins were detected using ECL chemiluminescence (Solarbio, Tongzhou, Beijing, China) and the Azure C300 chemiluminescence imaging system, and the Western blotting results were analyzed.

### 4.7. EdU Cell Proliferation Assay

The EdU cell proliferation assay was conducted in accordance with the protocol provided by the Cell-Light™ EdU Apollo 567 In Vitro Kit. A 48-well plate was pre-seeded with cells. Reagent A was diluted into the proliferation medium at a ratio of 1000:1 to achieve a final concentration of 50 μM EdU medium. Two hundred microliters of EdU medium was added to each well and incubated in a 37 °C, 5% CO_2_ incubator for 2 h. The EdU medium was removed, and the cells were subsequently washed 1–2 times with PBS. Subsequently, each well was treated with 200 μL of 4% paraformaldehyde (Solaibao, Beijing, China) for cell fixation at room temperature for 30 min. The fixative was removed, and each well was then treated with 200 μL of a 2 mg/mL glycine solution (Solaibao, Beijing, China) to neutralize the fixative, followed by a 5 min incubation on a decolorization shaker at 200 rpm at room temperature. The glycine solution was discarded, and the cells were subsequently washed with PBS once. Each well was then treated with 200 μL of PBS containing 0.5% Triton X-100 (Solaibao, Beijing, China) for cell permeabilization at room temperature for 10 min, after which the cells were washed once with PBS. Subsequently, 200 μL of 1× Apollo staining reaction solution was added to each well and incubated on a decolorization shaker at 200 rpm for 30 min at room temperature in the dark. The staining reaction solution was removed, and the cells were then washed 1–2 times with PBS. Each well was treated with 200 μL of ready-to-use DAPI solution and incubated at room temperature in the dark for 5 min, followed by 1–2 washes with PBS. Finally, 200 µL of PBS was added to each well, and images were captured using an inverted fluorescence microscope and counted using Image J software.

### 4.8. Cas9 Protein Purification

Total protein was extracted using non-denaturing WB/IP lysis buffer (Yeasen, Shanghai, China). Purified recombinant protein was obtained through affinity chromatography. The resin (Yeasen) was packed into an appropriate purification column by gravity, and the chromatography column was rinsed with 3–5 times the column volume of deionized water and then equilibrated with at least 5 times the column volume of Lysis Buffer. The sample was added to the equilibrated gravity column, and the retention time was at least 2 min to ensure sufficient contact between the target protein and Ni^2+^ ions, thereby improving the purification yield. The column was balanced with Wash Buffer, and the flow-through was collected. Elution was performed using 5–10 times the column volume of Elution Buffer, fractionally collected, with one tube per column volume, and each fraction was tested separately. Finally, the resin was washed sequentially with 3 times the column volume of Lysis Buffer and 5 times the column volume of deionized water. The purified protein was finally exchanged into the Cas storage buffer (580 mM KCl, 40 mM Tris–HCl, pH 7.5, 20% glycerol, 2 mM TCEP-HCl, and 2 mM MgCl_2_). The purified Cas protein was filtered through a 0.22 μm filter, aliquoted, and stored at −80 °C.

### 4.9. TAT-2.7 Cas9 Direct Delivery

In the experiment delivering TAT-Cas9-EGFP into the nucleus of the 293T cell line, 1 μg of TAT-Cas9-EGFP protein was mixed with 200 μL of fresh complete cell culture medium. The medium in the chamber slide, which was seeded with approximately 3000 cells the day before, was replaced with this mixed medium. After incubation in the incubator for 1 h, imaging was performed using confocal microscopy. Prior to this, an additional PBS washing step was carried out.

For the experiment of TAT-Cas9 delivery to the mChe reporter cell line, 400 ng of TAT-Cas9-EGFP protein in storage buffer was first mixed with 1 mL of fresh complete cell culture medium. The medium of the 24-well plate that was seeded the day before was replaced with the mixed medium, and liposome transfection was performed to deliver the mChe reporter plasmid. The cells were incubated in the incubator for 24 h and then subjected to FACS analysis. Prior to this, additional PBS washing step was carried out to remove any TAT-Cas9 protein bound to the cell surface.

### 4.10. Fluorescence Microscopy

Cells transfected with the pX458 plasmid were seeded into a 24-well plate, then stained with DAPI for approximately 15 min and washed three times with PBS for 5 min each. Images were captured using a fluorescence-inverted microscope (Nikon, Tokyo, Japan). The excitation light wavelength used for DAPI detection is 490 nm, for FITC detection it is 488 nm, and for TRITC detection it is 550 nm.

Cells delivered with TAT-Cas9-EGFP were seeded into chamber slides and stained with DAPI for approximately 15 min, followed by three washes with PBS, each for 5 min. Imaging was captured using a confocal laser scanning microscope (Nikon). The excitation light wavelength for DAPI detection was 490 nm, and for FITC detection, it was 488 nm.

### 4.11. Flow Cytometry

To evaluate the gene editing efficiency of TAT-Cas9-EGFP in the mChe reporter cells, the BD Accuri C6 Plus flow cytometer (BD Biosciences, Franklin Lakes, NJ, USA) was used to analyze the proportion of red fluorescence in 10,000 cells, in order to monitor the mChe-positive cell population over a period of time.

### 4.12. Statistical Analysis

All results are presented as the mean ± SEM. Statistical analyses of differences between groups were performed using two-tailed Student’s *t* test or chi-square test. * *p* < 0.05, ** *p* < 0.01, and *** *p* < 0.001.

## 5. Conclusions

In summary, this study employed PEG10 modification to develop a TAT-Cas9-EGFP site-specific integration cell line, which supports the stable expression of the TAT-Cas9-EGFP protein. This development lays the groundwork for the creation of a straightforward, efficient, well-tolerated, and low-toxicity CRISPR genome editing system capable of direct cellular delivery. Future research directions may include the assembly of ribonucleoprotein (RNP) complexes with the TAT-Cas9 protein, coupled with the use of PEG10-modified donor constructs, to facilitate efficient and stable site-specific integration.

## Data Availability

All data supporting the findings of this study are available in the article or in the Supplementary Data Files or are available from the corresponding author upon request. All raw data used in the study have been deposited at Figshare and are available at https://doi.org/10.6084/m9.figshare.27494436 (accessed on 13 December 2024).

## Supplementary Materials

The following supporting information can be downloaded at: www.mdpi.com/article/10.3390/ijms26031331/s1.
