# Supplementary material for: Construction of a TAT-Cas9-EGFP Site-Specific Integration Eukaryotic Cell Line Using Efficient PEG10 Modification
Source: Int J Mol Sci. 2025 Feb 4;26(3):1331. doi: 10.3390/ijms26031331 (PMC11818622; doi:10.3390/ijms26031331)
Supplement: Supplementary file 1 [file ijms-26-01331-s001.zip › ijms-3398494-supplementary.pdf]

Supplementary Materials

Table S1. The primers needed for the construction of TAT-Cas9-EGFP.

Primer name	Primer sequence（5‘-3’）
Cas9-F1	CGGATCCACTAGTCCAGTGTGGTGGGCCACCATGGACTATAAGGAC
Cas9-R1	TCGAGCGGCCGCCACTGTGCTGGATTTAATGGTGGTGATGATGGTGA
Cas9-F2	CCTGCGGCTAAGCGAGTCAAATTAGACGGTGGGTCACCGGCTGCAAAGCGCG<br>TCAAATTGGATGCTAGCGGCAGCGGCGACAAGAAGTACAGCATCGGC
Cas9-R2	AACTTTTCGTTTCTTTTTAGGATCCTCAACTTTTCGTTTTTTCTTAGGTGACCCGT<br>CGCCTCCCAGCTGAGACA
Cas9-F3	CCAGCGGCAAAAAGGGTGAAACTAGACGGGGGTAGCCCCGCCGCGAAGCGT<br>GTAAAGCTGGATGGAGGATCGCCTGCGGCTAAGCGAGTCAA
Cas9-R3	AACTTTTCGTTTCTTTTTAGGATC
Cas9-F4	AGACGACGACAGAGACGGAAGAAGAGGGGCGGCCATCCAGCGGCAAAAAGG<br>GTGAAA
Cas9-F5	GCCACCATGGACTATAAGGACCACGACGGAGACTACAAGGATCATGATATTGA<br>TTACAAAGACGATGACGATAAGATGGCCAGACGACGACAGAGACGGAA
Cas9-F6	CGGATCCACTAGTCCAGTGTGGTGGGCCACCATGGACTATAAGGAC
EGFP-F1	GATACCGGTGTGAGCAAGGGCGAGGAGCT
EGFP-R1	TTAATGGTGGTGATGATGGTGATGGTGGTGATGATGGTGGGAGCCGGAGCCGG<br>AGCCGAATTCCTTGTACAGCTCGTCCATGC
EGFP-F2	AGGATCCTAAAAAGAAACGAAAAGTTGATACCGGTGTGAGCAAGGG
EGFP-R2	TCGAGCGGCCGCCACTGTGCTGGATTTAATGGTGGTGATGATGGTGA
U6-F	TTTGCTGGCCTTTTGCTCACATGTGAGGGCCTATTTCCCATGATT
polyA-R	CATCACTAGGGGTTCCTGCGGCCGCTCCCCAGCATGCCTGCTATT

Table S2. The primers needed for the construction of U6-sgmChe-CBh-mcherry.

Primer name	Primer sequence（5‘-3’）
U6-F	TTTGCTGGCCTTTTGCTCACATGTGAGGGCCTATTTCCCATGATT
polyA-R	CATCACTAGGGGTTCCTGCGGCCGCTCCCCAGCATGCCTGCTATT

Table S3. Optimization of LipofectamineTM 3000 transfection conditions.

Liposomes /ul	Cas9/ ug	Donor /ug	Mass-to-volume ratio of DNA and liposomes	Mass ratio of Cas9 and Donor	Molar ratio of Cas9 and Donor
3.75	2.5		1：1.5		
7.5	2.5		1：3		
7.5	5		1：1.5		
3.75	1	1.5	1：1.5	1：1.5	1：1.375
3.75	0.5	2	1：1.5	1：4	1：10

Table S4. Editing efficiency detection primers.

Primer name	Primer sequence（5‘-3’）
T7EN 1F	GGCTACTGGCCTTATCTCAC
T7EN 1R	CATGCCGTCTTCACTCGCTG

Table S5. Primers for monoclonal identification.

Primer name	Primer sequence（5‘-3’）	The size of the amplified fragment
Left-F	CTGGCTCCATCGTAAGCAAACC	543
Left-R	CCCGTGAGTCAAACCGCTATCC	
Right-F	AGCCATACCACATTTGTAGAGG	520
Right-R	GTTCTCCTGTGGATTCGGGTCA	

Table S6. qPCR primer sequences.

Primer name	Primer sequence（5‘-3’）
β-Actin-F	AGATGTGGATCAGCAAGCAG
β-Actin-R	CCAATCTCATCTCGTTTTCTG
A2F	CAGAAACGCAGATCAAAGGGAG
A2R	TGGCAGGGCAGTTGGCGACCAT
BF	TTAGGAAGTGAAAGACGACCCA
BR	ACTACATTCTTTAGCGTGGTGA
NF	TCGTCCTACGGCTCTGTTAG
NR	GCTGGAGAGGCATCTGGGTG
PF	GCTGGAGCAGTTTGCCAAGGAC
PR	ACATCTTCCCATAGAGCGTGCC

Table S7. Targeting efficiency of Lin28A stable cell clones.

Targeted gene	Total clone number	5’ junction PCR positive clones (%)	3’ junction PCR positive clones (%)	positive clones(%)
Lin28A-Linear	25	9(36%)	7（28%)	7(28%)
Lin28A-PEG10	39	23(59%)	21（53%)	21(53%)

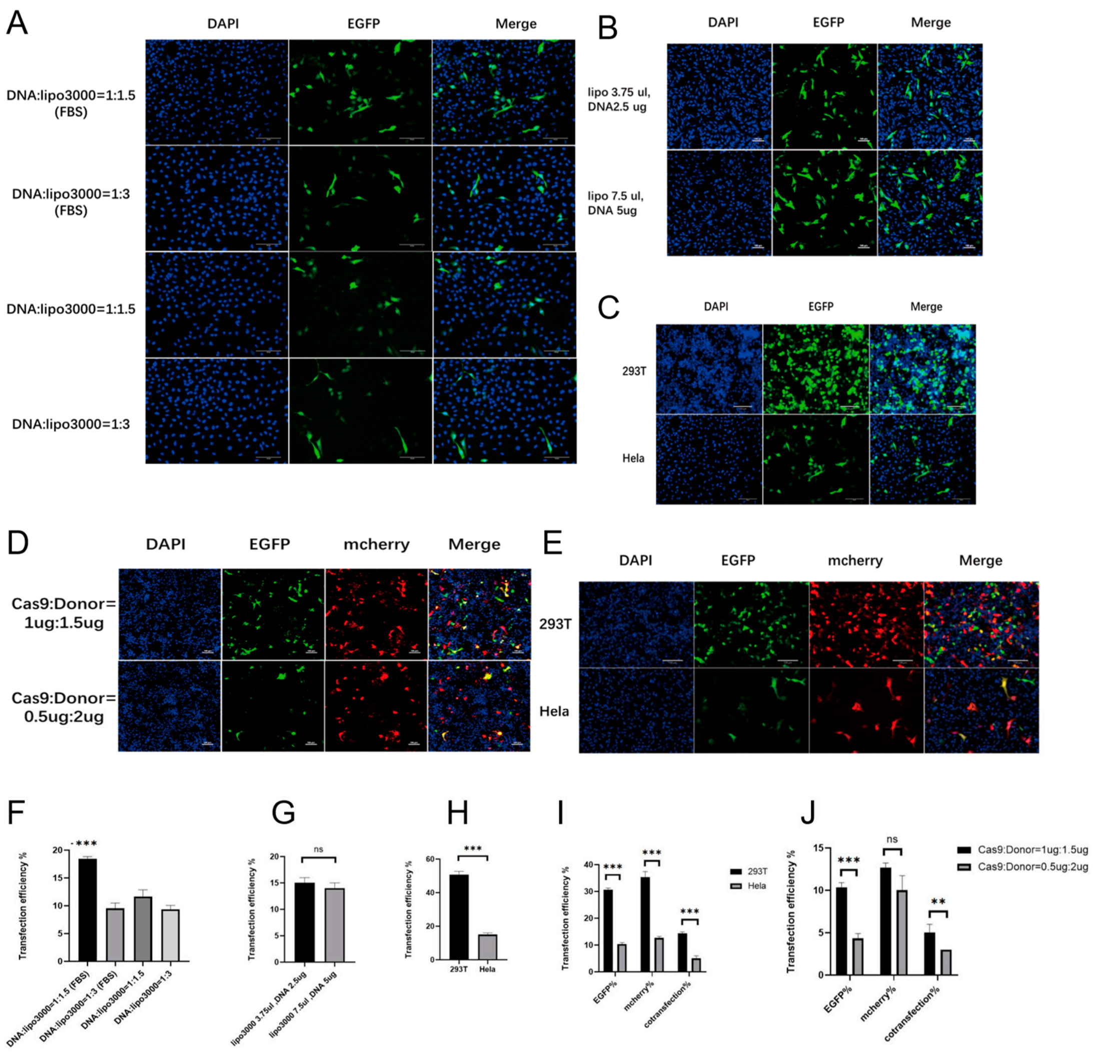

Figure S1. Optimization of transfection conditions. (A) shows fluorescence microscopy observations of Hela cells transfected with circular plasmid pX458 at different DNA to liposome ratios under various culture conditions. Scale bar 100 μ m. (B) shows fluorescence microscopy observations of Hela cells transfected with different amounts of circular plasmid pX458 and liposomes. Scale bar 100 μ m. (C) shows fluorescence microscopy observations of Hela and 293T cells transfected with circular plasmid pX458. Scale bar 100 μ m. (D) shows fluorescence microscopy observations of Hela cells transfected with circular plasmid pX458 and linear repair template pmcherry-N1-Linear at different mass ratios. Scale bar 100 μ m. (E) shows fluorescence microscopy observations of Hela and 293T cells co-transfected with

circular plasmid pX458 and linear repair template pmcherry-N1-Linear. Scale bar 100 μ m. (F) shows the impact of DNA to liposome ratio on the transfection efficiency of Hela cells. (G) shows the impact of DNA and liposome dosage on the transfection efficiency of Hela cells. (H) shows the impact of transfecting circular plasmid pX458 on the transfection efficiency of Hela and 293T cells. (I) shows the impact of co-transfecting circular plasmid pX458 and linear repair template pmcherry-N1-Linear on the transfection efficiency of Hela and 293T cells. (J) shows the impact of the ratio of circular plasmid pX458 and linear repair template pmcherry-N1-Linear on the co-transfection efficiency of Hela cells. Note: significance was calculated using t-test. *** indicates P<0.001, ** indicates P<0.01, and ns indicates P>0.05.

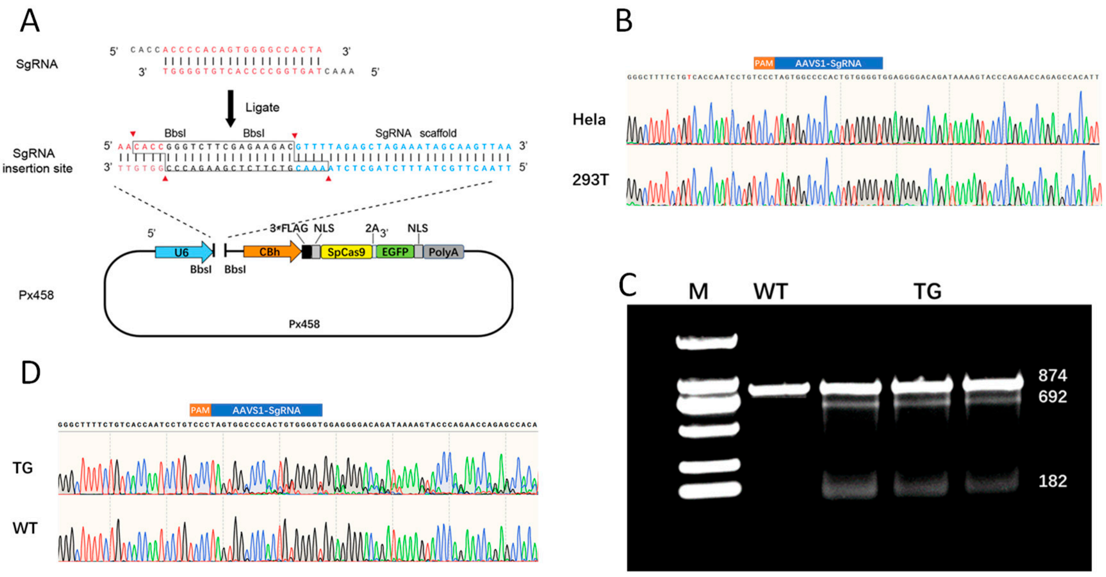

E

Binding sites				SgRNA guding sequence and base position																											Indel
sgRNA	Chromosome	Location	Direction	1	2	3	4		5	6	7	8	9	10	11	12	13	14		15	16		17		18	19	20	P	A	M	
ON	chr19	55115758	-	A	C	C	C		C	A	C	A	G	T	G	G	G	G		C	C		A		C	T	A	G	G	G	6/27
OT-1	chr1	2092860	+	A	C	C	A		C	A	C	A	G	T	G	G	G	C		C	C		A	G	C	T	A	G	G	G	0/24
OT-2	chr2	205620685	+	A	C	C	C		C	A	C	A	G	T	G	G	G	A		C	C		A	A	C	A	A	T	G	G	0/27
OT-3	chr7	157865490	+	A	C	C	C	T	C	A	C	C	G	T	G	G	G	G		C	C		A		C	G	A	C	G	G	0/24
OT-4	chr4	163472466	+	A	C	C	C		C	A	C	A	G	T	G	G	G	A	A	C	C		A		A	T	A	A	G	G	0/20
OT-5	chr22	47071498	-	A	C	C	C		C	A	G	A	G	T	G	G	G	G		C	C	G	A		C	T	C	A	G	G	0/25
OT-6	chr20	8412354	-	A	C	C	C		C	A	C	A	G	T	G	G	G	G	T	C	A		A		G	T	A	G	G	G	0/26

Figure S2. (A) The structure of pX458-sgRNA plasmid. (B) Assessment of sgRNA editing efficiency using the T7EN I cleavage assay. M, molecular weight standard; WT, wild-type cell genome; TG, transgene. (C) Nucleotide sequence analysis of the targeting site. (D) Detection of off-target sites in the AAVS1 site. (E) Detection of off-target sites in the AAVS1 site. OT, off-target. Green colors represent the same base at the target site; orange colors represent the same base at the PAM site.

A

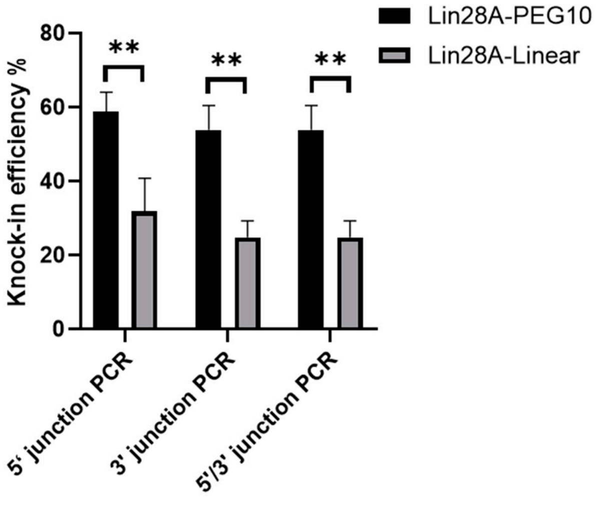

B

Targeting efficiency of stable cell clones

Targeted gene	Total clone number	5’ junction PCR positive clones	3’ junction PCR positive clones	Knock-in efficiency
Lin28A-PEG10	39	23(59%)	21(53%)	21(53%)
Lin28B-PEG10	36	20(56%)	19(53%)	19(53%)
Nanog-PEG10	43	23(53%)	21(49%)	21(49%)
PouV-PEG10	33	15(45%)	15(45%)	15(45%)

Figure S3. (A) KI efficiency of stable cell clones (cLin28A). The KI efficiency was obtained by the percentage of 5’/3’ junction PCR positive clones among the total clones investigated post-transfection, wherein we separately counted the ratios of positive clones at the 5’ end, 3’ end, and both sides of the junction to the total number of clones. n = 3 biological replicates; ** indicates P < 0.01. (B) The table of targeting efficiency of stable cell clones.

A

Targeted integration clones of Hela cells

Targeted gene	Total clone number	5’ junction PCR positive clones	3’ junction PCR positive clones	Knock-in efficiency
NP-PEG10	12	1(8.3%)	1(8.3%)	1(8.3%)
NP-linear	18	1(5.6%)	0(0)	0(0)

B

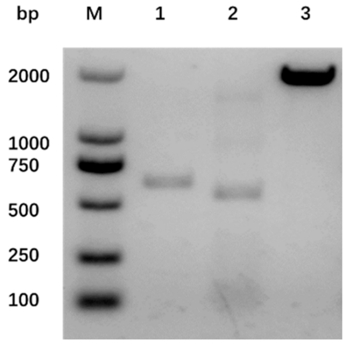

C

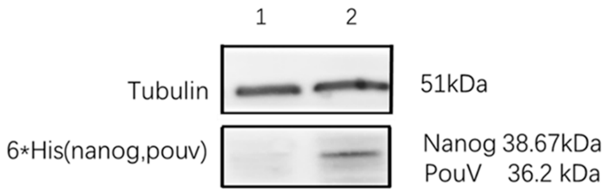

Figure S4. (A) The table of targeting integration clones of Hela cells. (B) Gel electrophoresis image. M, DNA molecular marker; 1, the result of the site-directed integration at the 5' end identified by Left-F/R; 2, the result of the site-directed integration at the 3' end identified by Right-F/R; 3, the result of the target gene identification of the NP cell line (C) Western blot

analysis of proteins expressed in insect cells. 1, wild-type Hela cells as control; 2, Nanog and PouV expression products.

A

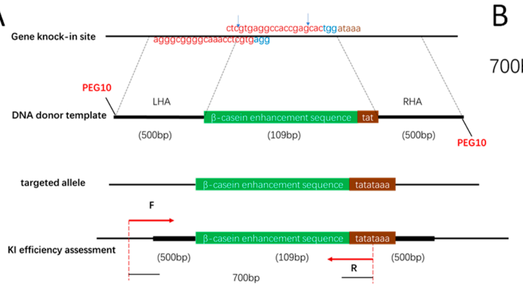

B

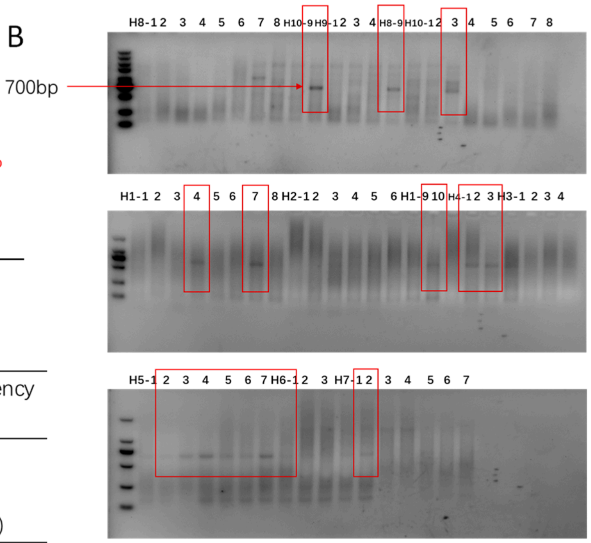

C

Targeted integration clones of GFF cells

Targeted gene	Total clone number	Knock-in efficiency
sgRNA	48	0(0/48)
sgRNA+PEG10	33	3.03%(1/33)
2sgRNA+PEG10	62	25.8%(16/62)

Figure S5. (A) Schematic diagram of the targeting strategy for the 500bp HA knock-in of lactoferrin regulatory region with PEG10-modified exogenous DNA fragment. The repair template sequence from 5' to 3' consists of: 5' homology arm sequence, β-casein enhancement sequence, and 3' homology arm sequence. (B) Electrophoresis diagram showing the insertion of the exogenous DNA template detected by PCR, with amplification bands of 700bp in size. (C) The table of targeted integration clones of GFF cells.
